# Radiation-Associated Fracture Nonunion of the Clavicle Treated with Locking Plate Fixation and Autologous Bone Grafting

**DOI:** 10.1155/2012/407349

**Published:** 2012-12-25

**Authors:** Takahiro Niikura, Sang Yang Lee, Yoshitada Sakai, Kotaro Nishida, Ryosuke Kuroda, Masahiro Kurosaka

**Affiliations:** Department of Orthopaedic Surgery, Kobe University Graduate School of Medicine, 7-5-1 Kusunoki-cho, Chuo-ku, Kobe 650-0017, Japan

## Abstract

We describe a case of radiation-associated fracture nonunion of the clavicle, which was treated by locking plate fixation and autologous bone grafting. The patient was a 67-year old man who received 70 Gy radiation therapy to treat nasopharyngeal carcinoma. Eight years later, he suffered a pathological fracture of the right clavicle. One year after the fracture, surgical treatment was performed due to persistent pain and weakness. Radiographs demonstrated atrophic nonunion. Bone scan demonstrated hot uptake at both ends of the fractured bone. MRI demonstrated a formation of pseudoarthrosis with fluid collection and suggested bone marrow edema at both ends of the fracture fragments. In surgery, fibrous pseudoarthrosis tissue was excised and both ends of the fracture fragments were refreshed to identify bleeding. Open reduction and internal fixation using a 7-hole locking plate and autologous bone grafting were performed. Successful bony union was obtained 1 year postoperatively, and no adverse events were observed up to 52 months after the operation. Our case suggests that a locking plate provides sufficient fixation and autologous bone grafting is effective in enhancing bone healing in a radiation-associated fracture nonunion of the clavicle in which it is difficult to achieve bony union.

## 1. Introduction

Radiation-induced complications in the mature bone include osteoradionecrosis, pathologic fracture, and radiation-induced neoplasms [1]. All types of cells can be injured or killed by radiation [1], and management of fractures caused by radiation is generally considered to be difficult due to the biologically inactive bone [2]. A high nonunion rate has been reported in the past literature [3, 4]. There are only two reports written in English that describe open reduction and internal fixation using a plate [5, 6]. Locking plate is a new technology, which provides excellent angular stability even in poor-quality bone, such as in osteoporosis patients, and can avoid the deterioration of periosteal circulation [7]. There has been no previous reports detailing the use of locking plate for the treatment of radiation-associated fractures of the clavicle. We report a case of radiation-associated fracture nonunion of the clavicle, which was treated by locking plate fixation and autologous bone grafting.

## 2. Case Report

The patient gave the informed consent prior being included into the study. The patient was a 67-year old man who received 70 Gy radiation therapy for the treatment of nasopharyngeal carcinoma. He received 20 Gy radiation therapy to the nasopharynx and 50 Gy to the supraclavicular fossa. He had concomitant hypothyroidism. He suffered osteoradionecrosis and osteomyelitis of the mandible and received hemimandibulectomy 6 years after the radiation therapy. He was also a smoker.

Eight years after the radiation therapy, he suffered a pathological fracture of the right clavicle without apparent trauma. The fracture was treated conservatively at an orthopaedic clinic; however, bony union was not obtained. The displacement increased over time, and he was referred to our hospital. One year after the fracture, surgical treatment was performed due to persistent pain and weakness.

**Figure 1:**
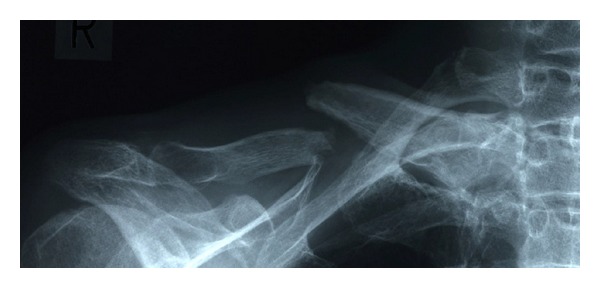
Preoperative radiograph of the fractured right clavicle demonstrating atrophic nonunion.

**Figure 2:**
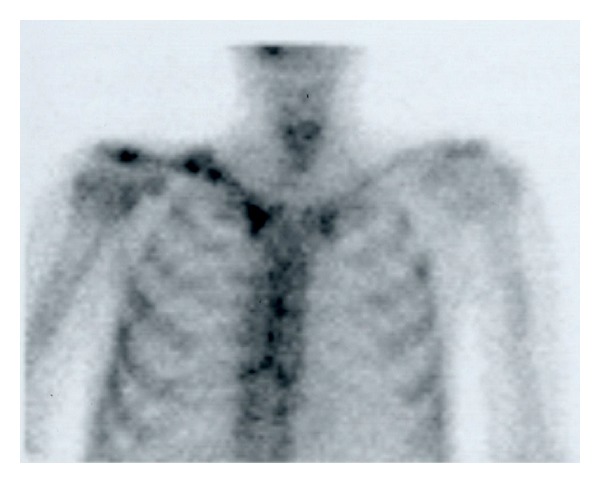
Preoperative bone scan demonstrating hot uptake at both ends of the fractured bone.

**Figure 3:**
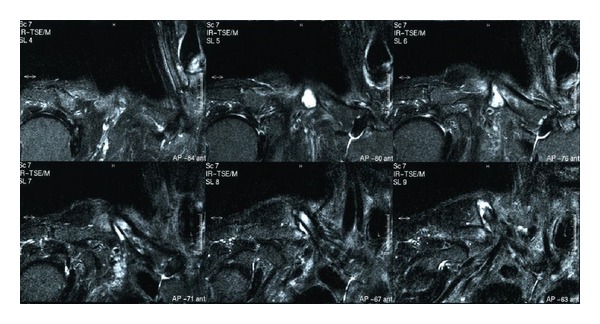
Preoperative MRI STIR images demonstrating a formation of pseudoarthrosis with fluid collection and suggesting bone marrow edema at both ends of the fracture fragments.

Radiographs demonstrated atrophic nonunion with displacement (Figure 1). Bone scan demonstrated hot uptake at both ends of the fractured bone (Figure 2). MRI demonstrated a formation of pseudoarthrosis with fluid collection and suggested bone marrow edema at both ends of the fracture fragments (Figure 3).

**Figure 4:**
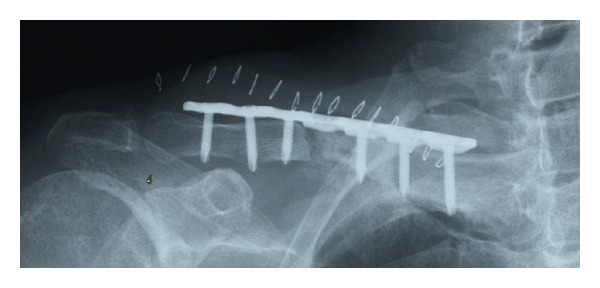
Postoperative radiograph demonstrating fracture fixation with 7-hole locking plate supplemented with autologous bone grafting.

In surgery, fibrous pseudoarthrosis tissue was excised and both ends of the fracture fragments were refreshed to identify bleeding. Open reduction and internal fixation using a 7-hole locking plate (Synthes) and autologous bone grafting were performed (Figure 4). Three locking screws were inserted into both fragments to fix the fracture. Cancellous bone harvested from the iliac crest was grafted to enhance bone healing.

**Figure 5:**
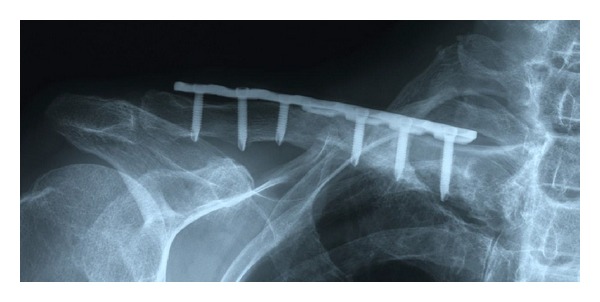
One-year postoperative radiograph demonstrating successful bony union.

Successful bony union was obtained by one year postoperatively (Figure 5). The pain disappeared and the range of motion fully recovered 6 months after the operation. The patient was satisfied with the treatment. No adverse events were observed and the reduction and fixation were maintained up to 52 months postoperatively. No further followup could be done due to the patient's death.

## 3. Discussion

Past reports suggest that fractures occur primarily with radiation doses >50 Gy [4, 8]. Our patient received 70 Gy radiation, and fracture occurred without apparent trauma; therefore, the fracture was diagnosed as a radiation-associated pathological fracture. Biopsy for pathology and culture are required for further investigation in these cases. The nonunion rate of radiation-associated fractures is reported as high at around 50%, and healing times tend to be long in fractures that eventually heal [3, 4]. The fracture developed atrophic nonunion radiographically. Difficulties in achieving bony union were predicted by the radiographic appearance. On the other hand, hot uptake at both ends of the fracture fragments was observed by a bone scan, and the finding suggested the existence of some biological bone activity. We hypothesized that the MRI finding of bone marrow edema suggested some biological bone activity even though the finding might be considered to show postirradiation change in the bone. However, because biological activity is considered to decrease as a result of radiation, we tried to achieve bony union by osteosynthesis with autologous bone grafting. If the bone activity was totally eliminated by radiation, vascularized bone graft should be necessary.

Due to the difficulty in obtaining bony union in radiation-associated fracture nonunions of the clavicle, past reports have recommended claviculectomy or resection of the pseudoarthrosis site [9, 10]. These procedures have been reported to be effective for pain relief and patient comfort. Only two clinical reports have described successful open reduction and internal fixation of radiation-associated clavicle fractures. Wera et al. reported on open reduction and internal fixation of radiation-associated clavicle fractures for 3 patients [5]. Two patients obtained bony union, and the remaining patient did not appear to achieve radiographic union but showed no loss of reduction at 28 months postoperatively. Fuchs et al. described the plate fixation and free vascularized corticoperiosteal bone graft for the treatment of radiation-associated fracture nonunion of the clavicle in two patients [6]. The fractures healed with good clinical results.

Wera et al. used autologous bone graft harvested from the iliac crest and demineralized bone matrix for the first patient, bone cement and allograft cancellous bone chips for the second patient, and demineralized bone matrix for the third patient as a void filler and enhancer of bone healing [5]. Fuchs et al. used a free vascularized corticoperiosteal bone graft from the medial femoral condyle to enhance bone healing [6]. We believe some supplementary bone healing enhancement is necessary and used autologous cancellous bone graft harvested from the iliac crest. Other tools enhancing bone healing, for example, bone morphogenetic proteins may also be useful; however, we consider that autologous bone grafting is better as it is osteogenic, osteoinductive, and osteoconductive. Vascularized bone graft is also a good option; however, it is technically challenging. Our treatment result suggests that autologous cancellous bone graft harvested from the iliac crest is sufficient for bony union. If a nonvascularized bone grafting fails, a vascularized bone grafting may become salvage [11].

Locking plate is a new technology which provides excellent angular stability and also preserves biology [7]. Irradiated bone results in a decrease in the circulation to bone and bone quality. Therefore, we think fixation using locking plate is superior to conventional plate fixation in both the biological and mechanical aspects. Locking plate fixation is thought to be a good choice for surgical treatment of radiation-associated fracture/nonunion of the clavicle.

## 4. Conclusion

Our case suggests that a locking plate provides sufficient fixation and autologous bone grafting is effective in enhancing bone healing in a radiation-associated fracture nonunion of the clavicle in which it is difficult to achieve bony union.

## References

[1] Mitchell MJ, Logan PM (1998). Radiation-induced changes in bone. *Radiographics*.

[2] Cannon CP, Lin PP, Lewis VO, Yasko AW (2008). Management of radiation-associated fractures. *Journal of the American Academy of Orthopaedic Surgeons*.

[3] Lin PP, Boland PJ, Healey JH (1998). Treatment of femoral fractures after irradiation. *Clinical Orthopaedics and Related Research*.

[4] Helmstedter CS, Goebel M, Zlotecki R, Scarborough MT (2001). Pathologic fractures after surgery and radiation for soft tissue tumors. *Clinical Orthopaedics and Related Research*.

[5] Wera G, Mohler DG, Chou L (2005). Surgical treatment of post-radiotherapy nonunions of the clavicle. *Bulletin: Hospital for Joint Diseases*.

[6] Fuchs B, Steinmann SP, Bishop AT (2005). Free vascularized corticoperiosteal bone graft for the treatment of persistent nonunion of the clavicle. *Journal of Shoulder and Elbow Surgery*.

[7] Wagner M (2003). General principles for the clinical use of the LCP. *Injury*.

[8] Holt GE, Griffin AM, Pintilie M (2005). Fractures following radiotherapy and limb-salvage surgery for lower extremity soft-tissue sarcomas: a comparison of high-dose and low-dose radiotherapy. *Journal of Bone and Joint Surgery A*.

[9] Spar I (1977). Total claviculectomy for pathological fractures. *Clinical Orthopaedics and Related Research*.

[10] Wang EHM, Sekyi-Otu A, O’Sullivan B, Bell RS (1992). Management of long-term postirradiation periclavicular complications. *Journal of Surgical Oncology*.

[11] Del Piñal F, García-Bernal FJ, Regalado J, Ayala H, Cagigal L, Studer A (2007). Vascularised corticoperiosteal grafts from the medial femoral condyle for difficult non-unions of the upper limb. *Journal of Hand Surgery*.

